# Development of mental health first-aid guidelines for psychosis: a Delphi expert consensus study in Argentina and Chile

**DOI:** 10.1186/s12888-024-05501-z

**Published:** 2024-02-09

**Authors:** Martín Agrest, Thamara Tapia-Munoz, Esteban Encina-Zúñiga, Isidora Vidal-Zamora, Norma Geffner, Sara Ardila-Gómez, Rubén Alvarado, Eduardo A. Leiderman, Nicola Reavley

**Affiliations:** 1 Proyecto Suma, Güemes 4130 (1425), Ciudad Autónoma de Buenos Aires, Argentina; 2 Universidad de Buenos Aires, Facultad de Psicología, Instituto de Investigaciones, Ciudad Autónoma de Buenos Aires, Argentina; 3 Department of Behavioural Science and Health, University College London, London, UK; 4 School of Public Health, Faculty of Medicine, Universidad de Chile, Santiago, Chile; 5 Department of Psychology, Faculty of Social Sciences, Universidad de Chile, Santiago, Chile; 6 Consejo Nacional de Investigaciones Científicas y Técnicas (CONICET), Ciudad Autónoma de Buenos Aires, Argentina; 7 Department of Public Health, School of Medicine, Faculty of Medicine, Universidad de Valparaíso, Valparaíso, Chile; 8 Departamento de Neurociencias, Facultad de Ciencias Sociales, Universidad de Palermo, Ciudad Autónoma de Buenos Aires, Argentina; 9 Centre for Mental Health, Melbourne School of Population and Global Health, University of Melbourne, Victoria, Australia

**Keywords:** Psychosis, Mental health first aid (MHFA), Cultural adaptation, Delphi study, Chile, Argentina

## Abstract

**Background:**

Psychotic symptoms may be less common than anxiety or affective symptoms, but they are still frequent and typically highly debilitating. Community members can have a role in helping to identify, offer initial help and facilitate access to mental health services of individuals experiencing psychosis. Mental health first aid guidelines for helping a person experiencing psychosis have been developed for the global north. This study aimed to adapt the English- language guidelines for Chile and Argentina.

**Methods:**

A Delphi expert consensus study was conducted with two panels of experts, one of people with lived experience of psychosis (either their own or as a carer; *n* = 29) and another one of health professionals (*n* = 29). Overall, 249 survey items from the original English guidelines and 26 items suggested by the local team formed a total of 275 that were evaluated in the first round. Participants were invited to rate how essential or important those statements were for Chile and Argentina, and encouraged to suggest new statements if necessary. These were presented in a second round. Items with 80% of endorsement by both panels were included in the guidelines for Chile and Argentina.

**Results:**

Data were obtained over two survey rounds. Consensus was achieved on 244 statements, including 26 statements locally generated for the second round. Almost 20% of the English statements were not endorsed (*n* = 50), showing the applicability of the original guidelines but also the importance of culturally adapting them. Attributions and tasks expected to be delivered by first aiders were shrunk in favour of a greater involvement of mental health professionals. Self-help strategies were mostly not endorsed and as were items relating to respecting the person’s autonomy.

**Conclusions:**

While panellists agreed that first aiders should be aware of human rights principles, items based on recovery principles were only partially endorsed. Further research on the dissemination of these guidelines and development of a Mental Health First Aid training course for Chile and Argentina is still required.

**Supplementary Information:**

The online version contains supplementary material available at 10.1186/s12888-024-05501-z.

## Background

While it has been estimated that narrowly defined non-affective psychotic disorders have a lifetime prevalence of 1.3% in the general population [1] and all psychotic disorders have a lifetime prevalence of 3% [2], subclinical psychotic experiences [3] or psychotic like experiences (i.e., subclinical delusional ideas and perceptual disturbances) [4] are far more common, with prevalence rising up to 5.8% [5] and even to 31.4% [6]). Moreover, psychotic symptoms of any kind are disturbing for the person experiencing them and their carers [7] and they can have persistent and debilitating effects (including, homelessness, unemployment, poorer physical health) [8].

Despite psychotic disorders being less common than other mental disorders (e.g., anxiety or affective disorders), they contribute significantly to the global burden of disease [9, 10] which, in addition to their early onset [11], usually makes them a high priority public health concern. Their early detection can contribute to a shorter duration of untreated psychosis (DUP), which has been associated with a better prognosis [12, 13]. In turn, timely professional help seeking can make a significant difference for individuals experiencing a first episode of psychosis, and this has been associated with various factors (including available and accessible health care services, lower levels of public and self-stigma levels, and support from family and community [14, 15]). However, early intervention for psychosis (EIP) services are still rare in many countries [16], and particularly in Latin America; a recent scoping review in the region was able to find only seven EIP programs and they were concentrated in just four countries (including Argentina and Chile) [17].

It has been estimated that in Latin America, a third of individuals experiencing non-affective psychosis do not receive any kind of treatment, from either general or specialized practitioners [18], a significant mental health treatment gap (albeit one that is lower than that for anxiety or alcohol misuse). Even where there are available services, many people experiencing psychosis may delay seeking help [19, 20]. Empowering community members to recognise and support a person in this situation may assist in earlier access to mental health services.

Increasing general population mental health literacy, through campaigns or more targeted interventions [21], may contribute to lowering stigma, may mitigate delays in seeking an initial healthcare consultation, and to increasing family and community understanding and helping behaviours towards individuals with mental health problems. Existing mental health services would then be more acceptable and accessible, ultimately contributing to recovery [22].

### Incidence and prevalence of psychosis in Chile and Argentina

The latest incidence study of narrowly defined non-affective psychosis in Chile showed that, between 2004 and 2017, there were 22,701 new confirmed cases and a 13.38 per 100.000 person-years incidence [23]. Significantly, Argentina lacks studies on either prevalence or incidence in the last 40 years with only one recent study on psychotic like experiences in Buenos Aires city, which showed a 18.0% prevalence among the general population [24].

### Mental health services for psychosis in Chile and Argentina

Chile and Argentina share a 5,300 km border and, importantly, some cultural traits (e.g., prevalence of Catholic traditions, importance of family bonds and friends) connected to the Spanish colonisation from XV to XIX century. Notwithstanding, with regards to health care services, Chile and Argentina have significant differences (e.g., in Argentina free access to a well-established health care network that includes mental health care for all kinds of mental health problems along with important budgetary constraints has led to salient mental health service gaps in Argentina [25]; while in Chile only selected health care problems are eligible for care with no out of pocket expenditure resulting in high standards of care and the incorporation of several mental health evidence based practices [26]).

After the military regimes that ruled both countries ended, in the middle 80s and in 1990, respectively, Argentina and Chile began a non-linear process of substituting long-term hospitalizations for community services. Despite notable progress towards community treatment of individuals with psychosis in both countries, community members’ awareness of the challenges arising from having psychotic symptoms as well as available services remains limited, and stigma stands out as major hurdle for their full social and economic inclusion [27, 28]. In line with international recommendations [29], improving mental health literacy, reducing stigma and developing helping skills among the general population may make a major contribution to earlier detection, improve recovery and higher levels of social and economic participation in these countries.

### Mental health first aid

The Mental Health First Aid (MHFA) training courses were developed to teach members of the public the needed skills to recognise when someone is developing a mental health problem (e.g., psychosis) or is in a mental health crisis (e.g., suicide) and to assist them by providing mental health first aid until the crisis is resolved or further health care is provided by health professionals [30]. The course is based on mental health first aid guidelines created using the Delphi expert consensus studies with people with lived experience of mental health problems and those who care for them, in addition to health care professional experts [31]. These Delphi studies to develop the guidelines were initially conducted with participants from Australia and other high-income English-speaking countries. The original English guidelines were also made available online for the public to access from the MHFA website (https://mhfa.com.au). More recently this initiative has spread to non-English speaking countries such as China, Sri Lanka, Brazil, Chile, and Argentina.

### Mental health first aid guidelines for psychosis

The first guidelines for helping individuals with psychosis developed by MHFA date to 2008 [32] and included 89 questionnaire items and 9 sections. An updated version of these guidelines was recently produced [33], and the guidelines now include 325 items and 17 sections. See Table 1 for the titles and examples of items included in each section.

**Table 1:**
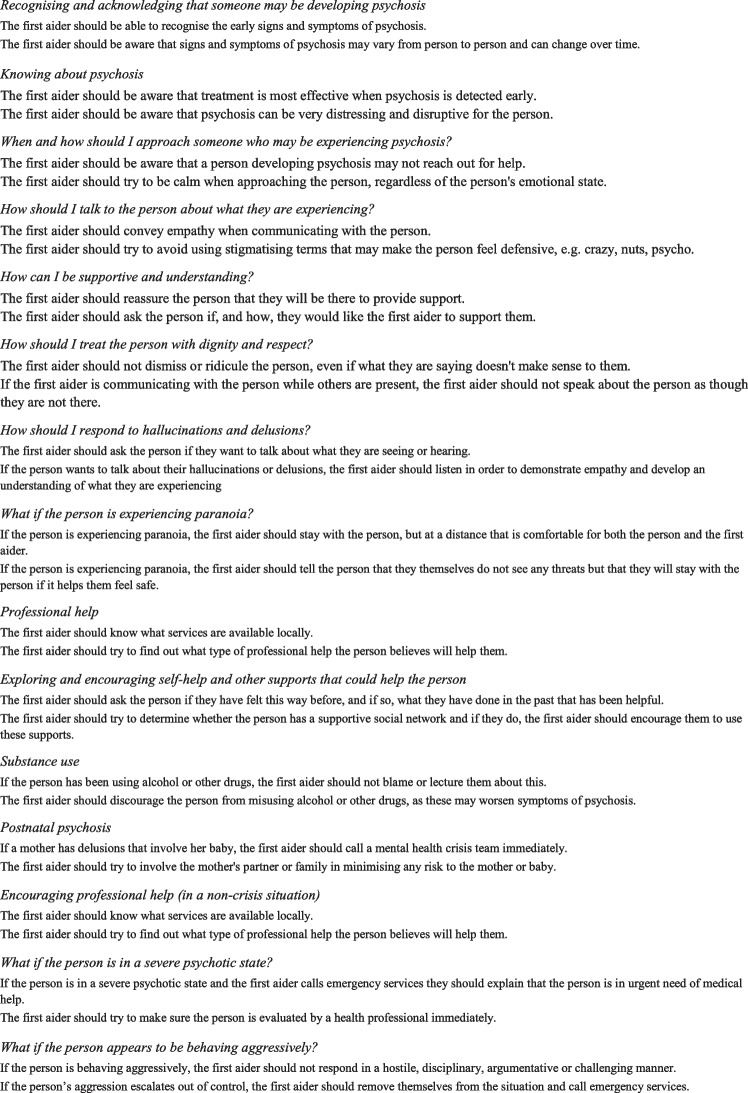
2019 Australian Mental Health First Aid guidelines for psychosis sections with example of items

Supporting the need for cultural adaption to the local context the recent adaptation for other contexts of MHFA guidelines for individuals experiencing psychosis showed key differences. In China, over 99% of the original English items evaluated by the local experts were endorsed for inclusion in the Chinese-language guidelines; eight new statements were also included, underscoring the importance of family involvement in the development of the Chinese-language guidelines [34]. Similarly, the Brazilian adaptation of these guidelines also emphasised the role of the family [35]. A previous study in Asia with only one panel (i.e., mental health clinicians from Cambodia, China, Hong Kong, Indonesia, Japan, Malaysia, Mongolia, Sri Lanka, South Korea, Taiwan, Thailand, and Vietnam) showed a lower rate of acceptance of the original items (51.1% endorsement rate) and a similar number of suggested new items (*n* = 8) [36].

This study aimed to use the Delphi expert consensus methodology to culturally adapt guidelines for lay members of the community interested in providing mental health first aid to someone experiencing psychosis in Chile and Argentina.

## Methods

As with the series of Delphi studies for culturally adapting the MHFA guidelines that have been conducted in other countries [34, 35, 37, 38], this study comprised the following four stages: (1) Development of the survey in Round 1; (2) Recruitment of experts for both panels of experts; (3) Data collection and analyses for the two rounds; and (4) Guidelines development.

### Development of the survey in round 1

The first-round questionnaire was developed by translating the statements that were included in the MHFA guidelines used in English-speaking countries to support a person with psychosis. The original items of the English guidelines were first translated into Spanish by a bilingual Australian native English speaker; secondly, the translation was reviewed by bilingual native Spanish speaker mental health professionals from Chile and Argentina to ensure a culturally pertinent translation. Twelve of these items were reformulated under the assumption of the research team that they would be better accepted after tailoring them to the local context. The result of this process was finally discussed with another member of the research team –who is a native English speaker (NR)– through back-translation of the modified items to ensure fidelity to the original version while respecting the cultural adaptation. An additional fourteen items were incorporated as part of the initial cultural adaptation including the generalisation to other non-crisis situations what the original guidelines focused only on that specific situation (e.g., taking seriously any threat posed by the person with psychosis, or being aware that the person can act based on their hallucinations, at any time they are experiencing psychosis and not just when they are in a crisis). A total of 249 items from the original English guidelines and 26 items suggested by the local team formed a total of 275 items, divided into 16 sections, reviewed by the two panels of experts. See Table 2 for examples of items and the name of the 16 sections.

**Table 2:**
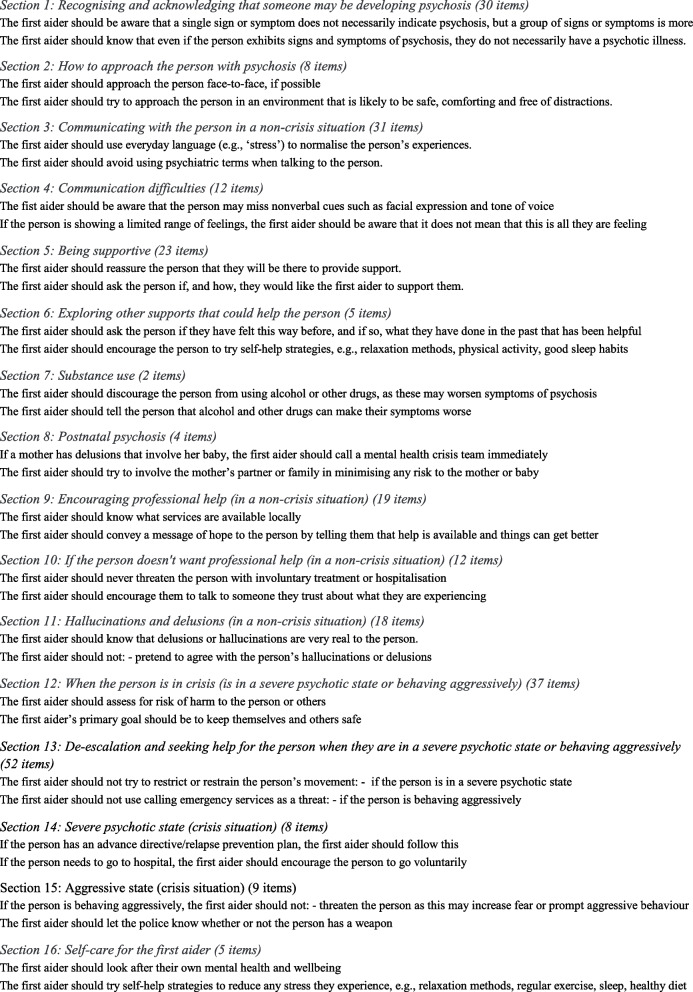
First round sections' name (number of items) and examples of items

### Recruitment of experts for both panels of experts

As in our previous studies [39, 40], the following criteria had to be met for a person to be an expert eligible for the study:

- Health professional expert panel members had more than four years of experience working as a healthcare professional with expertise and/or knowledge on psychosis. Eligible types of professions included, but were not limited to: general practitioners, psychiatrists, nurses, occupational therapists, psychologists, or social psychologists.
- Lived experience expert panel members self-identified as having experience with psychosis or caring for a person with psychosis.
- More than 18 years old.

Health professionals were recruited by the local research team familiar with key local experts with experience working with individuals with psychosis in different settings (e.g., inpatient units, day hospital, ambulatory care, rehabilitation). To ensure participant diversity, invited experts belonged to public and private institutions, worked in different cities, and had a variety of different approaches to mental illness. Personal invitations were sent by email or WhatsApp (a free US platform widely used for instant messaging between cell phones) with an explanation of the objectives of the study and the full information necessary for informed consent was also delivered. Less than 20% of invitations were declined, with most citing lack of time.

The lived experience panel was recruited through social media announcements by the University of Chile and through mental health professionals working with persons with a non-affective psychosis diagnosis who referred potential participants to the research team. After the initial contact, participants received a formal invitation with an explanation identical to that of health professionals and the same consent procedures were used.

This study began during the Covid-19 pandemic, so participants provided informed consent by email or WhatsApp. They signed the informed consent form with an image of their signature along with that of a witness.

### Data collection and analysis for the two rounds

Data for the first round was collected between March 11, 2020, and August 29, 2022. Data for the second round was collected between December 29, 2022, and May 17, 2023.

Using the same methodology as our previous studies [39, 40], the surveys collected participants' ratings of a set of statements on a 5-point Likert scale (1 = essential, 2 = important, 3 = unsure, 4 = not important, 5 = should not be included), choosing how important they considered the inclusion of each statement in the final mental health first aid guideline for psychosis in Argentina and Chile. In the first-round survey, at the end of each subsection or after each 10 items (whichever came first), open-text response boxes were displayed to allow participants to comment or suggest new items that they felt were important to incorporate into the final guidelines. MA and TT elaborated new items based on the suggestions from the first round.

Items were selected for the final guideline if at least 80% of the participants in both panels rated it as "essential" or "important". Meanwhile, statements rated as "essential" or "important" by 70.0—79.9% of the participants of at least one panel in the Round 1 survey were included in Round 2 for re-rating. Statements rated as "essential" or "important" by less than 70% of participants from at least one panel were immediately excluded from the final guideline. However, some items with an explanation for rejection in the comments were reformulated and presented in the second round. In Round 2, recommendations with an acceptance rate of at least 80% or more by one panel and at least 75% or more by the other panel were selected for the final guideline.

Spearman's correlation coefficient was estimated for the association analysis between the approval ratings of the professional and consumer panels. SPSS version 25 software was used.

### Guidelines development for Chile and Argentina

MA consolidated the recommendations from the two rounds of surveys into a preliminary guideline document. The rest of the team reviewed this draft version and made some comments. In parallel, these guidelines were sent to a small number of participants who explicitly expressed special interest in reviewing a preliminary version. No criteria for selection was used and every expert who volunteered to review the draft received a copy; only minor changes were included at this point that would not contradict the results of the Delphi process.

### Ethical approval

The study received ethical approval from the University of Melbourne (in Australia), the University of Palermo (Argentina) and the University of Chile (Chile).

## Results

**Fig. 1:**
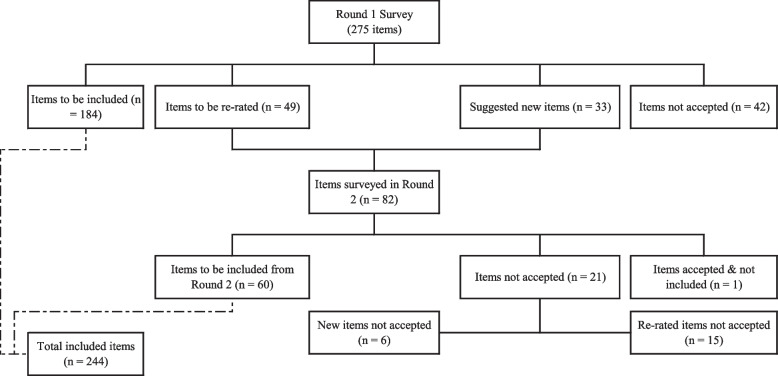
Overview of accepted and rejected items

From a total of 275 items rated in the first survey round and 82 items rated in the second round, 244 items were accepted for inclusion in the final guidelines. Figure 1 shows the overall process of including the statements through the two rounds.

### Round 1

A total of 58 participants completed the questionnaire in the first round of the Delphi study. The professional panel (*n* = 29) was unequally distributed between Chile (*n* = 10) and Argentina (*n* = 19) and included 14 psychiatrists, eight psychologists, three occupational therapists, two social workers, one nurse, and one researcher. The average years of experience as a health professional was 22.3 years; 48.3% were females (*n* = 14) and 51.7% were males (*n* = 15).

The lived experience panel (*n* = 29) also had more Argentinian participants (*n* = 23) than Chilean (*n* = 6). Sixteen were consumers and thirteen were caregivers and/or relatives. Of those who identified themselves as consumers in their primary role, two were also health professionals but in areas not related to mental health care; and of those who identified themselves as carers in the primary role, two were also health professionals. A total of 65.5% were females (*n* = 19) and 34.5% were males (*n* = 10). Lived experience participants were evenly distributed across age groups. Carers mostly belonged to the two eldest groups (55—64 and 65 or more years old). See Table 3 for a summary of the sociodemographic characteristics of participants.

**Table 3:**
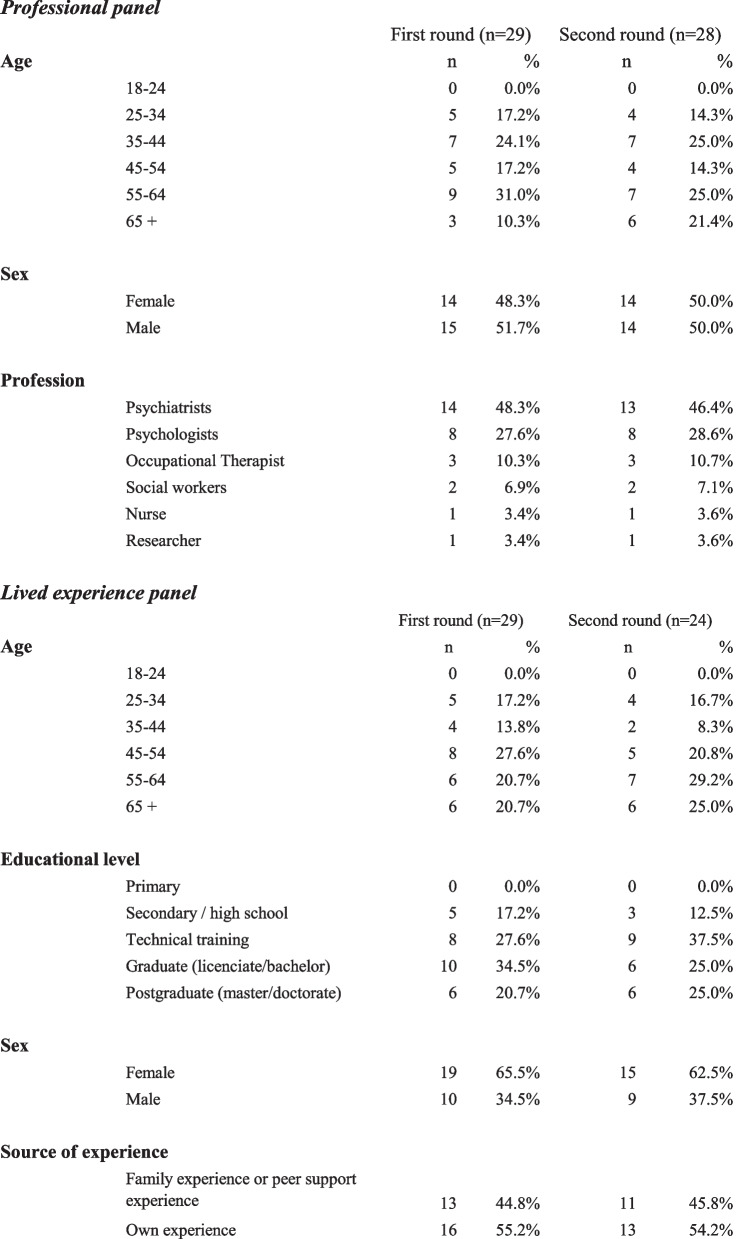
Characteristics of participants

Out of the 275 items included in the Round 1 survey, 184 items (66.9%) were endorsed as *essential* or *important* by 80% or more of the experts in both panels. Another 49 items (17.8%) required re-rating in Round 2, and 42 (15.3%) items were rejected (See Fig. 1).

### Round 2

The Round 2 questionnaire included 49 items to be re-rated and 33 new items suggested in Round 1. A total of 52 participants completed Round 2, with 28 participants from the health professional panel (response rate of 96.6%) and 24 participants from the lived experience panel (response rate of and 82.8%). No new participants were added in Round 2. Out of the 82 items rated in Round 2, 73.2% (*n *= 60) were endorsed by both panels and thus included in the final guidelines. Another 26.8% (*n* = 22) were not included (i.e., 21 items did not meet the inclusion criteria and one was excluded due to a different formulation of the item receiving greater approval).

### Differences between the Spanish-language guidelines for Chile and Argentina and the English-language guidelines

When comparing the English and Spanish guidelines, it was noted that 37 statements (14.9%) included in the English guidelines were not accepted by the Argentinian and Chilean experts in the first round. Another 13 statements from the English guidelines were not endorsed in Round 2, totalizing 20.1% of the original items (*n* = 50) not accepted to be part of the Psychosis local guidelines. Similarly, among the 59 items suggested by the research team and the local experts (i.e., 26 statements suggested before Round 1 and 33 statements suggested during Round 1 and tested in Round 2), 20.3% (*n* = 12) were finally discarded by the two panels of experts.

The rejected statements comprised all the original items regarding substance abuse and every reference to self-help strategies and healthy living styles. Additional rejected items pertained to recommendations to join support groups, several aspects regarding how to encourage professional help when the person is not in a crisis, and how to approach and to help a person in crisis in a severe psychotic state or behaving in an aggressive mode. See Table 4 for examples of rejected items.

**Table 4:**
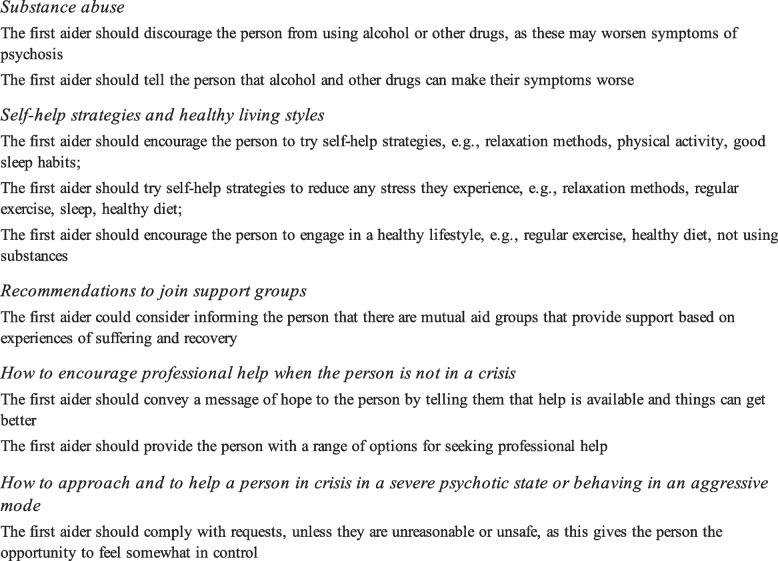
Rejected topics and items from the English guidelines

When developing the first-round survey, the research team included the option that some of the actions a first aider should do in Argentina and Chile would not apply only to when the person is in a severe psychotic state or behaving aggressively but also to any situation when the person is experiencing psychosis. The local experts rated almost all the items as essential or important in all situations in which the person is experiencing psychosis, not just those involving aggressive behaviour or severe psychosis, including those relating to taking threats seriously and when to contact emergency services. The only item that was endorsed solely for situations of aggression or severe psychosis was that relating to calling for professional assistance if unable to de-escalate the situation.

The statements with the lowest endorsement ratings from both panels were those relating to taking precautions when communicating with a person with psychosis only if they were in crisis or behaving aggressively. According to local experts, precautions are necessary when communicating with a person with any symptoms of psychosis.

Other statements receiving a low endorsement rate among the local experts included “The first aider should approach the person face-to-face, if possible” (with less than a 60% endorsement rate in both panels) and “The first aider should try to gather information about whether the person feels safe, e.g., by stating ‘You seem worried; is there anything I can do to help?’ or ‘Do you feel safe? Or is there something you are afraid of?’,” which was not accepted if the person was in a severe psychotic state or behaving aggressively. In addition, both panels rejected that “The first aider should ask the person if they are afraid or confused” when the person show signs of having hallucinations or delusions (although not in a crisis) and, also, if the person is experiencing paranoia, that “the first aider should ask the person about their fears.”

Interestingly, both panels rejected the idea that “The first aider should support the person in making their own decisions about their mental health” (endorsement rates: 65.5% and 58.6% in the lived experience and professional panel respectively).

### Similarities between panels

Over both rounds, experts from both panels had a high level of agreement (*r* = 0.66 in Round 1 and *r* = 0.56 in Round 2). A total of 68.0% of the statements (*n* = 144) in Round 1 had less than a 10% difference in the percentage of panel members endorsing those items, including 15.6% with an absolute agreement (*n* = 43).

Agreement was high for several key sections, notably “Recognising and acknowledging that someone may be developing psychosis”, and “How to approach the person with psychosis.” (See Table 5).

**Table 5:**
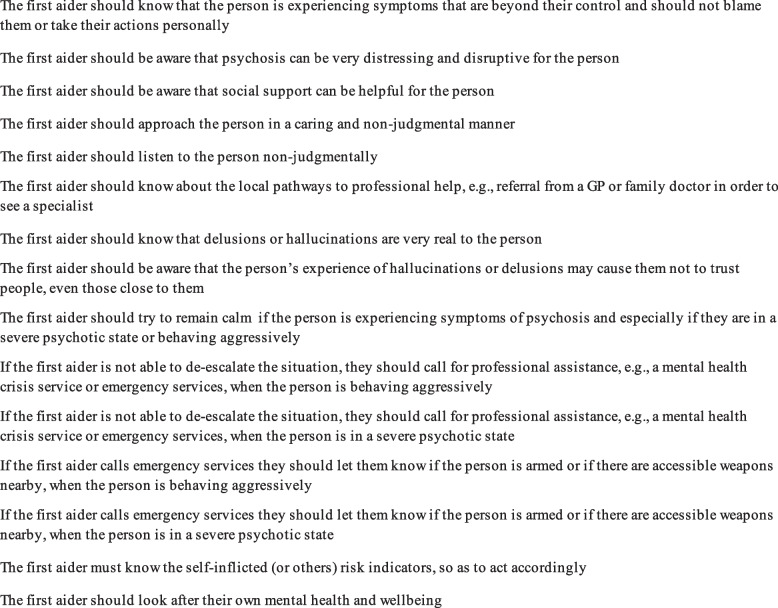
Statements unanimously endorsed by both panels of experts

There were also similarities with regards to rejected statements, including: The first aider should not try to restrict or restrain the person’s movement if the person is in a severe psychotic state (or behaving aggressively); The first aider should avoid using patronising or trivialising statements when interacting with the person, e.g., ‘cheer up’, ‘I’m sure it will pass’ and ‘it could be worse’.

### Differences between the lived experience and health professional panels

Despite a general high level of agreement between panels, for 2.2% of the statements (*n* = 6) the difference between the percentage of panel members endorsing those items was 30% or more. The statement with the largest endorsement difference between panels was “If the person agrees to seek professional help, the first aider should encourage them to request a longer appointment so they will have adequate time to discuss their symptoms and concerns” (75.9% lived experience panel vs. 27.6% professional panel). Other statements with a significant difference between panels were related to self-help strategies and healthy living styles. According to the lived experience panel these were important messages that the first aider could convey (both items received a 78.6% of endorsement). However, the professional panel was critical of suggestions for other strategies beyond specialty care for individuals with psychosis (i.e., these items were endorsed by only 37.9% and 44.8% of the members in the professional panel).

The expert panels had a significant disagreement with regards to how the first aider can be supportive to the person by “continue to reach out to the person, e.g., to let the person know they are thinking about them and that they care” (79.3% endorsement rate among the lived experience panel and 44.8% among professionals). In the same vein, both panels disagreed on what the first aider should do if the person is experiencing paranoia (although not in a crisis): According to the lived experience panel it would be acceptable that the first aider “encourage and support the person to move away from whatever is causing their fear, if it is safe to do so,” while the professional panel rejected this alternative (endorsement rates were respectively 79.3% and 55.2%).

See supplementary file 1 for details of the ratings of statements by round and panel, and supplementary file 2 for the final guidelines text in Spanish.

## Discussion

The present study aimed to use the Delphi expert consensus method to culturally adapt guidelines for community members wishing to provide mental health first aid to someone experiencing psychotic symptoms in Chile and Argentina. This was achieved by a two-round Delphi survey, involving mental health professionals, people with lived experience and carers. Mental health first aid original actions for individuals with psychosis were mostly endorsed by the local experts. However, actions relating to substance misuse, self-help strategies and healthy living styles were not recommended by the Chilean and Argentinian panellists, mostly due to lower endorsement by the professional panel.

### No help other than professional help

Local experts consistently preferred mental health professionals to assist a person with psychotic symptoms over self-help and mutual-help strategies (e.g., consumer led support groups for gradual discontinuation of medication). The latter strategies were largely not endorsed by the professional panel and were just below the endorsement cutoff among the lived experience panel. It is possible that such strategies were seen as less than the optimal help for individuals with psychosis. Interestingly, professionals also rejected the importance of suggesting the existence of education and employment programs, while the lived experience panel endorsed this statement; but this may have been due to the still very limited availability of such programs in the region –as far as the professional experts are aware of them.

Similar low endorsement rates by Chilean and Argentinian experts for self-help strategies were also seen in Delphi Studies to develop guidelines for other conditions (e.g., depression, alcohol consumption) [39, 40]. Local health professionals do not appear to be confident about the value of self-help strategies, which may pose an additional hurdle to the implementation of internationally accepted initiatives based on what individuals can do for themselves (e.g., Self-Help Plus [41], Illness Management and Recovery [42–44]). Furthermore, the item about the first aider supporting the person in making their own decisions about their mental health was also rejected. This probably points to professionals' limited recognition of the importance of autonomy among individuals with psychosis.

In the same vein, local experts were reluctant to endorse statements about making suggestions with regards to the person being aware of the dangerousness of substance use in the context of experiencing psychosis –despite accepting that substance misuse could be a factor triggering psychosis. Overall, health professional experts were less prone to the first aider giving advice on substance use, despite other studies have suggested that substance use could be a significant trigger for individuals with psychosis relapses [45, 46]. This apparent contradiction could be due to their lack of confidence in the capacity of individuals with psychosis to follow such advice and to their opinion that this should be addressed in the context (and as part) of a mental health treatment.

Attributions and tasks expected to be delivered by first aiders were shrunk in favour of a greater involvement of mental health professionals, particularly by the professional panel; in comparison, the lived experience panel was more open to accepting a wider involvement of lay members of the community after they are trained. Such disagreement sheds light on the reluctance of mental health professionals to accept any other non-specialist involvement in the care of individuals with mental health problems; similar issues were found during the analysis of the implementation of mhGAP in the region (more notably in Argentina, than in Chile [47, 48]). In addition, the disagreement between panels of experts further underscores the value of also considering the perspectives of people with lived experience, in mental health research broadly and particularly in relation to how they wish to be supported by people in their social networks.

### Communicating with caution not just for individuals in crisis

Being cautious when communicating with a person experiencing psychosis was not seen as applying only to situations where the person is in a severe psychotic state or behaving aggressively. Furthermore, there was consensus that the first aider should not only call the emergency services if the person was in a severe condition or had a weapon but could do this any time they felt insecure. According to the local experts, any person with psychosis could potentially misunderstand communications and easily turn the interaction with the first aider into a risky situation, pointing to the need for safety measures despite the person’s reliability and full personhood being jeopardised because of them. Understanding of recovery principles is still limited in the region [49, 50], which, along with limited awareness of the Convention on the Rights of Persons with Disabilities [51] among many Latin American experts, may be leading to limited acknowledgement of self-determination, lack of acceptance of “the dignity of risk” [52], and relatively low value placed on the importance of instilling hope among individuals with psychosis. This may be illustrated by the finding that, while lived experience experts endorsed conveying “a message of hope to the person by telling them that help is available and things can get better”, health professional experts did not consider that this message could be beneficial to the person with psychosis.

### Mental health first aid: challenges and opportunities for Chile and Argentina

Despite a general acceptance of what a first aider should do to help a person with psychosis and a general adequate alignment with the Chilean and the Argentinian mental health laws and action plans [53–56], several challenges may still remain in order to gain greater acceptance of MHFA training, particularly from local mental health professionals. Chile has greater experience with evidence-based interventions aiming to help individuals with psychosis which could facilitate buy-in of MHFA for this population. In turn, Argentina has a long tradition celebrating the participation of community members and involving lay persons in helping others in need –which has been decisively promoted during the last two decades. However, while the original guidelines are based on (and take for granted) the recovery orientation of mental health care services and professionals, both Chile and Argentina need to further transition from a paternalistic understanding of caring for individuals with psychosis to a more respectful attitude that incorporates a focus on self-determination [57]. Civil legislation affecting mental health workers would contribute to this situation by putting pressure on them to take full responsibility for anything their patients might do in the community. Furthermore, media reporting of mental illness [58], along with health care workers opposing recovery tenets and worried families that do not have access to adequate community services, reinforce fears and doubts with regards to people with psychosis –posing severe challenges to developing non-professional help for these persons.

In summary, stigma towards individuals with psychosis, largely acknowledged in Chile [59, 60] and Argentina [28], has multiple implications for the lives of people experiencing psychosis and leads to a potential role for MHFA guidelines and training to tackle this. A review of mental health stigma research in Argentina showed an increased interest in this topic following the enactment of the 2010 national mental health law [61]. However, this body of research shows that anti-stigma initiatives (including short films, lived experience testimonies, and television shows) have been insufficient to substantially modify these attitudes. Implementing MHFA training in key settings in which people are more likely to have contact with people with mental health problems could complement anti-stigma and recovery initiatives and contribute to concrete ways of fighting stigma [62]. Educational settings (e.g., elementary and high school) and emergency services (including police officers), are likely to be particularly suitable settings in which to begin implementation.

### Strengths and limitations

A key strength of this study is a research design that gives equal weight to the views of health professionals and people with lived experience. This is relevant considering that the objective was to culturally adapt the recommendations, an issue that is unlikely to be achieved only with input from professionals.

The significant number of changes introduced to the original guidelines supports the importance of this cultural adaptation. Compared to other adaptation studies, Chile and Argentina stood out for a relatively low endorsement rate of the original items and a larger number of suggested statements.

In terms of limitations, the absence of full back-translation of all the original English statements may have affected the comparison of accepted and rejected items in our study and the Australian study [33]. In addition, participants were mainly from metropolitan areas, which limits the generalisability to rural areas, although a variety of metropolitan areas were included (e.g., Jujuy, Concordia, La Plata, Buenos Aires, in Argentina). Additionally, experts were not equally distributed by country; Argentina contributed more experts than Chile in both panels. However, the presence of participants from both Chile and Argentina supports the case for generalisability of the findings to other Latin American Spanish-speaking countries.

Further studies are needed relating to implementation of the guidelines to fully explore their potential for domestic use in Latin America.

## Conclusion

A Delphi expert consensus study involving health professionals and people with lived experience was used to adapt the mental health first aid guidelines for psychosis for Chile and Argentina. The adapted guidelines preserved most of the original guidelines, but trimmed down self-help strategies and emphasised the need to count on mental health specialists for a wider range of situations when a person is having psychotic symptoms. Human rights information was added to the first aider’s toolbox while recovery principles were partially endorsed. Further research on dissemination, acceptance, training, and usage of the guidelines in Chile and Argentina is pending for these countries.

### Supplementary Information

**Additional file 1. **

**Additional file 2. **

## Data Availability

The data supporting our findings is attached as Additional file 1, which contains all the statements that were presented to the panels and their endorsement rates.

## Abbreviations

DUP: Duration of Untreated Psychosis
EIP: Early Intervention for Psychosis
LMICs: Low- and middle-income countries
MHFA: Mental Health First Aid
