# Supplementary material for: Development of mental health first-aid guidelines for psychosis: a Delphi expert consensus study in Argentina and Chile
Source: BMC Psychiatry. 2024 Feb 9;24:113. doi: 10.1186/s12888-024-05501-z (PMC10858466; doi:10.1186/s12888-024-05501-z)
Supplement: Supplementary file 2 — Additional file 2. [file 12888_2024_5501_MOESM2_ESM.docx]

***Supplementary File 2 – Spanish Guidelines for Psychosis***

**Cómo brindar ayuda a una persona con síntomas de psicosis**

**Guía de primeros auxilios en salud mental**

En esta guía encontrarás un conjunto de recomendaciones para ayudar a una persona que experimenta síntomas de psicosis. Estas recomendaciones están divididas en diferentes secciones: Cómo reconocer que alguien puede estar desarrollando psicosis, Cómo acercarse, Cómo comunicarse si la persona no se encuentra en crisis, Dificultades y recaudos en la comunicación, Cómo brindar apoyo, Explorando sobre situaciones que podrían ayudar a la persona experimentando psicosis, Cómo alentar la ayuda profesional y qué hacer si la persona no quiere ayuda profesional en una situación sin crisis, Cómo manejarse si la persona está teniendo alucinaciones o delirios aunque no esté en crisis, Qué hacer en situaciones de crisis, Cómo cuidar la propia salud mental.

Al finalizar podrás ver detalles de cómo se realizó esta guía y sugerencias para su utilización o consultas adicionales.

*Qué son los síntomas de psicosis*

Psicosis es un término general para describir un problema de salud mental en el que una persona pierde el contacto con la realidad. Es común como parte de la psicosis el que se produzcan alteraciones significativas del pensamiento, las emociones y el comportamiento. La psicosis puede alterar la vida de una persona, dificultando el inicio o el mantenimiento de relaciones, el cuidado personal, el trabajo u otras actividades habituales, y afectando su calidad de vida y la de sus familiares.

Los síntomas de la psicosis pueden estar presentes en diferentes problemas de salud mental, como la esquizofrenia, el trastorno bipolar o la demencia, o pueden ser fenómenos transitorios o que se relacionan con el consumo de alcohol u otras drogas, o también ser el producto de traumatismos cerebrales u otras enfermedades físicas.

Es importante señalar que algunas personas que experimentan psicosis no la considerarán un problema, y también que en algunos casos no genera situaciones de crisis (como serían un estado psicótico grave, tener pensamientos o comportamientos extremos). La agresividad puede ocasionalmente relacionarse con fenómenos psicóticos, aunque es más frecuente que sean víctimas de situaciones de violencia que perpetradores de violencia.

Esta guía sirve para prestar primeros auxilios de salud mental a una persona que ha sufrido un impacto negativo por su experiencia de psicosis. Salvo que se indique lo contrario, la información de las recomendaciones de esta guía se refiere a situaciones que no son de crisis.

**Reconocer que alguien puede estar desarrollando psicosis**

Es importante que puedas reconocer los signos y síntomas tempranos de psicosis. Por ejemplo, los cambios abruptos en la forma de ser de la persona, la falta de motivación o interés de la persona en la vida, pueden ser signos tempranos de psicosis, aunque es importante saber que éstos pueden variar de una persona a otra y que también pueden cambiar con el tiempo. Un solo signo o síntoma no necesariamente indica psicosis, pero es más probable que un grupo de signos o síntomas sí lo sea. Éstos pueden aparecer repentinamente o desarrollarse gradualmente con el tiempo. Sin embargo, si aparecen gradualmente (o no están claros) tampoco deberías ignorarlos. No deberías asumir que los signos y síntomas de psicosis desaparecerán por sí solos ni que probablemente se trata de que la persona está "pasando por una fase", experimentando los altibajos normales de la vida o que son producto del uso indebido de sustancias. Si se trata de una persona joven tampoco debes asumir que los cambios en el funcionamiento o comportamientos extraños que se repiten son "solo una etapa" o "algo de adolescentes". Aun así, debes saber que incluso si la persona presenta signos y síntomas de psicosis, no necesariamente tiene un trastorno psicótico. En principio, los síntomas de psicosis pueden estar presentes en una serie de trastornos mentales (no únicamente en personas a quienes los profesionales de la salud mental diagnosticarían con un trastorno psicótico). En ocasiones, una persona puede experimentar psicosis como un episodio único o como parte de una enfermedad en curso, como la esquizofrenia, el trastorno bipolar o la demencia. Incluso tratándose de un trastorno, la persona puede experimentar múltiples episodios de psicosis con períodos de bienestar intermedios. Asimismo, los síntomas de psicosis pueden estar relacionados con el uso de drogas o alcohol.

Existen una variedad de factores que pueden desencadenar la psicosis (por ejemplo, abuso de sustancias, estrés extremo o trauma). Si bien los síntomas de psicosis pueden ser desencadenados por el uso de sustancias, el consumo de drogas y alcohol no necesariamente tiene como consecuencia síntomas de psicosis.

Es importante que sepas que la psicosis puede ser muy angustiante y perturbadora para la persona, así como también para quienes la rodean. La psicosis puede tener un gran impacto en la vida cotidiana de la persona, sus relaciones, el trabajo o el estudio. Sin embargo, debes saber que la persona puede ser consciente de lo que le está sucediendo o puede no tener idea de lo que le pasa o puede costarle aceptar que no se encuentra bien. Es posible, entonces, que la persona no busque ayuda. Además, hay personas que pueden esconder o disimular signos o síntomas de psicosis, lo que puede hacer más difícil darse cuenta de lo que le pasa.

Debes ser respetuoso de las creencias espirituales/religiosas y de las características culturales de la persona.

Es importante que sepas que la psicosis no es contagiosa y tampoco es una discapacidad intelectual. No debes culpar a la persona por lo que le sucede ni tomar sus acciones personalmente ya que los síntomas que experimenta están más allá de su control. Debes ser consciente de que puedes sentir una variedad de emociones (por ejemplo, conmoción, confusión o culpa) al darte cuenta de que alguien cercano está experimentando síntomas de psicosis, y que estas son reacciones comunes.

Es importante que sepas que el apoyo social puede ser útil para la persona, así como también que la persona puede carecer de este apoyo porque se hubiera aislado o porque otros se hubieran alejado de ella por no comprender su comportamiento.

La falta de información y los prejuicios en la sociedad pueden hacer más difícil la atención y recuperación de la persona. Asimismo, deberías tener en cuenta tus propias ideas o prejuicios respecto de la psicosis porque esto puede afectar la manera como te relacionas con la persona. Es conveniente que tengas información respecto a temas de derechos humanos. Adicionalmente, podrías informarte sobre la Ley de Derechos de los Pacientes (Ley 20.584 en Chile; Ley 26.529 en Argentina).

El tratamiento es más efectivo cuando la psicosis se detecta tempranamente. Por tal motivo, ante la duda de si la persona está experimentando psicosis, debes consultar a un profesional de salud mental para recibir asesoramiento.

*Signos y síntomas comunes cuando una persona está desarrollando psicosis*^1^

Cambios afectivos y/o de motivación: Depresión, ansiedad, irritabilidad, suspicacia, afectividad aplanada o inapropiada; cambios en el apetito; energía y motivación reducidas.

Cambios en el modo de pensamiento y la percepción: Problemas de concentración o atención; sentido de alteración de sí mismo, de los otros o del mundo exterior (por ejemplo, sentir que uno mismo o que los otros han tenido cambios inexplicables o que la explicación resulta incomprensible); ideas extrañas; experiencias perceptivas inusuales (por ejemplo, reducción o aumento de la intensidad del olfato, el sonido o el color que no se explica por otra condición de salud).

Cambios en el comportamiento: Alteraciones del sueño; aislamiento o retracción social; reducción en la capacidad para desempeñarse en el trabajo o en roles sociales.

^1^ Adaptado de: Edwards, J & McGorry, PD (2002). Implementing Early Intervention in Psychosis. Martin Dunitz, London.

**Acercarse a la persona**

Debes tratar de acercarte a la persona en un entorno que sea seguro y libre de distracciones, y en el que la persona pueda sentirse cómoda. Debes tomarte el tiempo necesario para tener una conversación con la persona, sin que ésta pueda vivirlo como algo apresurado. Trata de mantener la calma cuando te acerques a la persona, independientemente de su estado emocional. Intenta hacerlo de manera cuidadosa y sin juzgarla, sin recurrir a una manera confrontativa.

Al acercarte a la persona presta atención y ten en cuenta la forma en que ésta se comporta (por ejemplo, si la persona está desconfiada y evita el contacto visual, debes ser sensible a esto y darle a la persona el espacio que necesita).

**Comunicación (en una situación sin crisis)**

Para una persona que experimenta síntomas de psicosis puede no ser fácil animarse a contar lo que está viviendo. Muchas veces, y sobre todo en momentos iniciales, resulta difícil para la propia persona comprender lo que le está pasando; otras veces, la expectativa es ser rechazada, criticada o mal tratada. Es habitual que la sociedad mire con prejuicio a quien tiene un padecimiento mental y especialmente a quien tiene síntomas de psicosis. La comunicación, aun fuera de una situación de crisis, debe sortear este primer obstáculo que involucra el temor y el alejamiento de la gente respecto de quien experimenta psicosis. El miedo a ser estigmatizado y algunas creencias ya internalizadas sobre la psicosis pueden afectar la comunicación.

*Estigma y auto-estigma*

Las personas con síntomas de psicosis se enfrentan con frecuencia a los prejuicios, las emociones negativas y las conductas de rechazo de su comunidad. Este fenómeno social lleva el nombre de estigma, una marca que genera una devaluación de las personas ante la mirada del resto, tomándose una parte o aspecto de una persona como si la definiese completamente, y que solo por un rasgo entonces se espera que esa persona tenga una serie de características no deseadas.

Luego de ver y escuchar estas actitudes en la comunidad algunas personas interiorizan esta forma de pensar, sentir y actuar, respecto de personas con alguna condición de salud mental, llegando a verse a sí mismas de igual forma negativa que lo hace la sociedad.

Es fundamental transmitir empatía cuando te comunicas con la persona; usar un lenguaje cotidiano (por ejemplo, "estrés") para normalizar sus experiencias, y tratar de evitar el uso de términos estigmatizantes que pueden hacer que la persona se sienta a la defensiva (por ejemplo, loco/a, chiflado/a, psicoseado/a, etc.). Puede ser útil evitar el uso de términos que puedan hacer pensar a la persona que lo que le sucede es algo permanente (por ejemplo, que “es” de cierta forma en vez de decir que “está” de cierta forma).

Otros ingredientes importantes de la comunicación con una persona que experimenta psicosis y no está en crisis son: escuchar a la persona sin juzgarla, hacerle saber que está siendo escuchada (por ejemplo, puedes demostrar tu atención repitiendo o retomando algo que la persona haya dicho), prestar atención a la comunicación no verbal de la persona (aspecto, gestos, miradas, posturas y actitudes) a fin de mejorar la comunicación.

Una buena estrategia para hablar con la persona suele ser tratar de aproximarse de a poco al problema que ella tiene, y gradualmente desarrollar preguntas más específicas sobre lo que la persona está experimentando.

Incluso si la persona se da cuenta de que no se encuentra bien, su confusión y temor acerca de lo que le está sucediendo pueden llevarla a negar que le estuviera pasando algo malo. Ten presente que la persona puede estar asustada por sus pensamientos y sentimientos. Debes ser consciente de que pueden ser necesarias varias conversaciones antes de que la persona pueda hablar acerca de lo que le pasa. Debes preguntarle a la persona si quiere hablar sobre cómo se siente, e intenta permitirle hablar sobre sus experiencias, sentimientos y creencias si ésta así lo desea.

Ten en cuenta que la persona puede ser vaga al describir sus síntomas y puede enfatizar los síntomas físicos sobre los síntomas de una enfermedad mental. Otras veces la persona puede reaccionar con emociones que no parecen encajar en el contexto de la conversación (por ejemplo, riendo) producto de la angustia o de aspectos de su malestar que aún no hubiera mencionado. Si la persona está angustiada por sus experiencias, debes preguntarle qué la ayudaría a sentirse más segura o tranquila.

Es importante evitar ser negativo o pesimista al hablar con la persona sobre su futuro y no hagas especulaciones con la persona sobre un diagnóstico. Trata de mantenerte en calma cuando estés con la persona y evitar, por ejemplo, estar moviendo las piernas, cambiando de posturas o mordiéndote las uñas. Las pausas y los silencios están bien y, si bien puede resultar incómodo, la persona puede necesitar que la acompañen en silencio. Si en algún caso el comportamiento de la persona te resulta molesto o irritante, debes entender que la situación puede ser angustiante para ambos y conservar la calma. Recuerda considerar tomarte el tiempo necesario para responder las preguntas que tenga la persona.

Qué hacer si la persona niega que algo esté mal o no desea hablar sobre lo que está experimentando:

- Trata de centrarte en escuchar en lugar de tratar que la persona cambie de opinión;
- Pregúntale si hay algo específico que puedas hacer para ayudarla;
- Dile que estarás disponible para hablar en el futuro;
- Evita discutir con la persona.

**Dificultades en la comunicación**

Debes ser consciente de que las dificultades en la comunicación son frecuentes en general (y no siempre entendemos lo que la otra persona quiere decir o la otra persona no siempre entiende lo que queríamos decir). En situaciones de psicosis esto puede incrementarse. La persona que experimenta psicosis puede no interpretar o malinterpretar señales no verbales, como la expresión facial y el tono de voz; y, puede no ser capaz de comunicarse de la forma en que normalmente lo haría (por ejemplo, respondiendo con respuestas no relacionadas o desviándose de un tema a otro). Debes ser consciente de que la persona puede estar escuchando voces, que nadie más oye, lo que dificulta la comunicación. También debes evitar el uso de lenguaje complejo (por ejemplo, metáforas o sarcasmos), pero sin subestimar la capacidad de comprensión de la persona. No debes asumir que la persona no puede entender lo que se le está diciendo, incluso si su respuesta es limitada.

Asimismo, debes permitirle a la persona el tiempo suficiente para responder a preguntas o afirmaciones, ya que puede tener dificultades para procesar la información.

Recuerda tratar de comunicarte de forma clara y sencilla y repetir las cosas cuando sea necesario.

Si es necesario que te acerques o hacer contacto físico con la persona, debes pedirle permiso a la persona, por ejemplo, diciendo: '¿te importa si me siento a tu lado?' O 'Puedo ver que te duele el brazo, ¿está bien si uso el botiquín de primeros auxilios para vendarlo? '. Si es apropiado y factible, debes consultar con otras personas que conozcan a la persona para obtener consejos sobre la mejor manera de comunicarte con ella.

Debes tratar de regular el nivel de emoción que muestras al hablar para no generar reacciones extremas en la persona. Y, siempre recuerda que debes tratar de no tomar personalmente nada de lo que la persona te diga, aunque te resulte ofensivo o alejado de tus opiniones.

**Brindando apoyo**

A la hora de brindar tu apoyo lo primero a considerar es que siempre debes tratar a la persona con respeto y no debes descartar o ridiculizar a la persona, incluso si lo que ésta dice no tiene sentido para ti. Si hablas con la persona mientras otros están presentes, no debes hablar de la persona con estos otros como si ella no estuviera allí.

Luego, es importante que le preguntes a la persona si le gustaría recibir tu apoyo, y de qué forma. Debes intentar asegurarle a la persona que estarás allí para brindarle el apoyo que ella necesite y para tratar que ella no corra riesgos. Pregúntale a la persona si existen factores estresantes actuales que puedan estar contribuyendo a cómo se está sintiendo o a lo que le está pasando, y si le gustaría recibir apoyo práctico (por ejemplo, para llamar a un familiar o ayudarla con alguna cuestión doméstica inmediata). Es importante que le dejes en claro lo que podés y estás dispuesto a hacer para apoyarla, así la persona sabe de tus capacidades y también de tus límites.

Considera decirle a la persona que entiendes que pueda estar asustada por lo que está experimentando. Es una buena idea reconocer que la persona debió movilizar sus propios recursos (coraje, inteligencia, buen juicio) para abrirse a hablar o aceptar ayuda, especialmente por el estigma social que suele recaer sobre personas con psicosis. Es importante validar los sentimientos de la persona en cualquier instancia; incluso si la persona cree en cosas que no son reales, sus sentimientos sí lo son.

En ninguna circunstancia debes amenazar a la persona en un intento por hacerla cambiar su comportamiento y tampoco deberías contradecirla (sin por ello tener que mentirle). Recuerda que es posible que la persona no pueda seguir ninguna sugerencia que le hagas.

Algunas personas con psicosis pueden tener dificultades de expresar lo que sienten y tomar de modo muy literal lo que se les dice.

Es importante que tengas presente que puede ser necesario que estés disponible y/o debas brindar información a familiares o a personas cercanas de personas si te lo piden. Sin embargo, al mismo tiempo recuerda que debes respetar la privacidad de la persona y su derecho a la confidencialidad (a menos que la persona esté en riesgo para sí misma o para otros). Si la persona es un adolescente, y la situación lo requiere, debes ofrecerle quedarte con éste mientras se comunican y hablan con un familiar u otro adulto de confianza.

Si tienes contacto continuo con la persona, debes estar atento a los signos que indiquen que la persona puede estar experimentando un empeoramiento de sus síntomas.

Siempre que te sea posible intenta trabajar en red con los apoyos comunitarios y los servicios de salud/ salud mental. Brindar apoyo no debería ser una tarea solitaria.

**Explorando sobre situaciones que podrían ayudar a la persona experimentando psicosis**

El uso de otros apoyos no especializados (además de los psiquiatras, psicólogos y otros trabajadores de la salud, o de la familia) no han sido muy recomendados por los expertos locales. Tampoco han sido recomendados localmente las estrategias de auto-ayuda (métodos de relajación, etc.), aunque lo son en otros países del mundo, sobre todo si no hay disponibilidad de profesionales de la salud. De todos modos podrías tratar de determinar si la persona tiene una red social de apoyo y, si la tiene, alentarla a usar estos apoyos. Además, deberías preguntar a la persona si se ha sentido de esta manera antes, y si es así, qué ha hecho en el pasado que le hubiese sido útil para ver si puede recurrir nuevamente a esta opción.

**Uso de sustancias**

Las recomendaciones sobre la exploración y las sugerencias referidas al uso de sustancias en personas con síntomas de psicosis no alcanzaron un consenso entre los expertos locales, por más que se sabe que el uso de sustancias podría tener un impacto en el inicio o en las recaídas de un cuadro de psicosis. Sin embargo, sí es importante que en caso de preguntar a la persona por conductas de consumo de sustancias lo hagas sin juzgarla. También puedes tener presentes las guías de primeros auxilios referidas al consumo de alcohol que se han adaptado localmente.

**Psicosis postparto**

La psicosis postparto es la presencia de síntomas de psicosis poco después de haber dado a luz y pueden involucrar tanto alucinaciones como delirios (frecuentemente relacionados con el bebé). Esta es una situación particular dentro de la experiencia de síntomas de psicosis y las recomendaciones también son especiales. Si crees que una madre puede estar experimentando psicosis postparto, debes llamar a un equipo de crisis de salud mental de inmediato, ya que el problema puede escalar rápidamente y las demoras en el tratamiento pueden aumentar el riesgo para la madre y su bebé. Especialmente, si una madre tiene delirios que involucran a su bebé, debes llamar a un equipo de crisis de salud mental con absoluta premura y asegurarte de que alguien esté con ella en todo momento hasta que reciba ayuda profesional.

Trata de involucrar a la pareja o la familia de la madre para minimizar cualquier riesgo para la madre o el bebé. Si bien debes prestar apoyo inmediato y contactarte con los equipos especializados, es importante que también puedas escuchar a la madre.

**Alentar la ayuda profesional (en una situación sin crisis)**

*Qué trabajadores de la salud mental pueden ayudar a una persona con psicosis*

Existe una variedad de profesionales y colaboradores que según el lugar pueden (o no) estar disponibles. Cada uno de ellos tiene un tipo de preparación diferente y utiliza recursos que pueden tener sus particularidades.

Médicos generalistas

Son personas que han estudiado la carrera de Medicina y tienen conocimientos generales sobre salud y enfermedad. Su comprensión más amplia puede ser una buena puerta de ingreso para recibir atención especializada y, en algunos casos, solo se accede a la atención especializada si se recibe una derivación de su parte.

Médicos psiquiatras

Son personas que han estudiado la carrera de Medicina y luego se han especializado en la rama de la Psiquiatría. Si bien pueden tener conocimientos generales sobre salud y enfermedad se han especializado en los trastornos mentales. Cuentan con la posibilidad de recetar medicamentos además de contar con la posibilidad de brindar tratamientos psicológicos, así como lo pueden hacer otros profesionales de la salud mental.

Psicólogos

Son personas que han estudiado la carrera de Psicología y suelen tener una aproximación al padecimiento mental por medio de la escucha y la intervención a través de la palabra. Suelen desarrollar tratamientos psicológicos, los cuales pueden tener una variedad de enfoques u orientaciones (como el psicoanálisis, la terapia cognitiva, la terapia gestáltica, la terapia dialéctico comportamental, entre otras alternativas).

Terapeutas ocupacionales

Son personas que han estudiado la carrera de Terapia Ocupacional. Algunos de estos profesionales se han especializado en ayudar a personas con trastornos mentales (aunque otros pueden desempeñarse en ámbitos por completo diferentes, como en Centros de Rehabilitación física o respiratorios). Su enfoque suele ayudar con las habilidades y actividades de la vida diaria a través de la escucha, el entrenamiento o la supervisión de tareas específicas en base a fines consensuados con la persona.

Trabajadores sociales

Son personas que han estudiado la carrera de Trabajo Social. Suelen tener un enfoque que incluye las dinámicas sociales que rodean la recuperación de personas con padecimiento mental y, entre otras tareas, están especialmente capacitados para colaborar con la gestión de viviendas, trabajos, beneficios sociales, etc.

Enfermeros

Son personas que han estudiado la carrera de Enfermería y se han especializado en temas de salud mental. En general se desempeñan dentro de ámbitos asistenciales generales o especializados. Suelen tener gran experiencia en acompañar en situaciones cotidianas y contener emocionalmente a las personas.

Acompañantes terapéuticos

Son personas que han realizado estudios específicos para acompañar a personas en situaciones de crisis para evitar internaciones por temas de salud mental en centros específicos o para acompañar la transición luego de una internación. Suelen trabajar en equipo con profesionales de la salud mental y/o con otros acompañantes terapéuticos. En algunos casos es un paso previo a obtener el título de Licenciado en Psicología, aunque en otros es finalmente el trabajo en el que se especializan.

Psicólogos sociales

Son personas que han estudiado la carrera técnica superior en Psicología Social y se ocupan de las formas de interrelación de las personas en los grupos (dinámica grupal), así como de los vínculos, las comunicaciones y las interrelaciones que se generan entre los individuos cuando se agrupan con un objetivo común. Su trabajo suele ser eminentemente en el campo de los grupos humanos.

Trabajadores comunitarios

Son personas sin estudios técnicos o profesionales en temas de salud mental, aunque sí tienen experiencia en la realización de tareas de acompañamiento en la comunidad.

Acompañantes personales

En el marco de la Convención por los Derechos de las Personas con Discapacidad, un agente de salud reconocido es quien provee el apoyo necesario para posibilitar la inclusión social de personas con alguna discapacidad. El acompañante personal es una persona que ha realizado una capacitación específica en temas de discapacidad y brinda los apoyos que una persona con discapacidad puede necesitar (por ejemplo, alguien con discapacidad psicosocial requiere acompañamiento para tareas en la comunidad y el acompañante personal se lo provee para que la persona no cuente con desventajas respecto de otras personas sin esa discapacidad).

Pares especializados

Son personas que ayudan a partir de su propia experiencia de padecimiento y recuperación en el campo de la salud mental. En ocasiones se desempeñan dentro de instituciones tradicionales o pueden hacerlo de forma independiente o junto a profesionales de la salud mental. Es un recurso no tradicional de ayuda aunque su desarrollo sería incipiente en Chile y Argentina.

*Qué tratamientos existen para personas con psicosis*

Los tratamientos posibles para la psicosis son múltiples. Por lo general, las personas con psicosis pueden ser ayudadas con tratamientos especializados que ofrecen los psiquiatras, los psicólogos y los terapeutas ocupacionales (de modo individual o combinadamente) sin ser necesario un tratamiento institucional. La participación social a través de grupos de pertenencia, la realización de actividades con sentido y/o el trabajo o el estudio, suelen ser importantes para el bienestar de todas las personas y eso incluye, por supuesto, a quienes desarrollan síntomas de psicosis. Los tratamientos especializados suelen intentar promover justamente estas actividades y pueden hacerse más intensivos (o con apoyo institucional) cuando éstas se ven interferidas. Idealmente las actividades que promueven la recuperación de personas con psicosis deben hacerse en la comunidad y no dentro de instituciones de salud mental. Sin embargo, en algunas ocasiones se vuelve difícil acompañar a las personas en la comunidad y se recurre a los tratamientos que ofrecen las instituciones de salud mental o los servicios de salud mental en instituciones de salud general. Excepcionalmente las personas con psicosis pueden requerir internaciones que, en caso de ser necesarias, no suelen durar más que algunos días o pocas semanas. Las recomendaciones internacionales sugieren el privilegio de internaciones en centros de salud general (polivalentes) por sobre instituciones monovalentes o “psiquiátricas”. Sin embargo, por fuera del ámbito público las internaciones por temas de salud mental suelen cursar en instituciones monovalentes. Y, en el ámbito público, las instituciones polivalentes no siempre cuentan con la infraestructura requerida para este tipo de internaciones. Durante las internaciones, aunque no solo durante éstas, ocupan un rol muy importante el personal de enfermería, los trabajadores sociales y, algunas veces, también los acompañantes terapéuticos junto a los psiquiatras y psicólogos.

La medicación es un recurso valioso que puede evitar crisis y permitir el desempeño de tareas en la comunidad sin la interferencia de los síntomas de psicosis. La psicoterapia se ha visto que puede ayudar a reducir el impacto de los síntomas y permite a la persona afrontar mejor situaciones que contribuyen a la exacerbación de los síntomas de psicosis. Existen diferentes formas de psicoterapia que pueden ser de ayuda en estos casos y cuentan con evidencia de ser útiles: el tratamiento cognitivo conductual suele ser la forma de psicoterapia con mayor evidencia a nivel internacional, pero también hay experiencias efectivas de abordajes basados en el psicoanálisis, la terapia de apoyo, la terapia familiar, la terapia grupal, la terapia dialéctico comportamental, etc. La terapia ocupacional suele desempeñar un rol muy importante como parte de la rehabilitación de personas con psicosis, especialmente para quienes han visto reducida su participación social.

Cada lugar tiene sus características particulares en cuanto a disponibilidad y accesibilidad a servicios de salud generales y especializados. En primer lugar, debes saber qué servicios están disponibles localmente, tener algún conocimiento general sobre los tipos de tratamiento que pueden ser útiles para la psicosis (ver recuadro), estar al tanto de la diferencia entre los profesionales de salud mental (psiquiatra, psicólogo/a, trabajador/a social, enfermero/a, terapeuta ocupacional, etc.) y sus roles respectivos (ver recuadro), y conocer las vías locales para obtener ayuda profesional (por ejemplo, si hace falta la referencia de un médico de cabecera o un médico de familia para consultar a un especialista, o lugares que brindan ayuda para estos casos).

Debes decirle a la persona que lo que está experimentando podría mejorar con la ayuda profesional adecuada y es importante que le comuniques que buscar ayuda profesional lo antes posible es importante para evitar que los síntomas empeoren, aunque deberías hacerlo sin presionarla para que busque ayuda. Debes asegurarle a la persona que está bien buscar ayuda y señalar que buscar ayuda es un signo de fortaleza en lugar de un signo de debilidad o fracaso. No utilices un enfoque amenazador o confrontativo cuando alientas a la persona a buscar ayuda profesional. Pregúntale a la persona si tiene un médico de confianza y, si lo tiene, aliéntala a buscar ayuda profesional de su médico (ya que un médico de cabecera / un médico clínico o generalista puede ser un buen punto de contacto cuando busque ayuda profesional). Si la persona tiene dudas sobre la posibilidad de ser internada, es conveniente explicar a la persona que buscar ayuda profesional no necesariamente significa que será hospitalizada, ya que el tratamiento temprano puede llevarse a cabo de manera ambulatoria.

Es importante que seas consciente de la influencia que puede tener la familia de la persona respecto de la búsqueda de ayuda (por ejemplo, la familia puede alentar o desalentarla a obtener la atención que necesita). Teniendo en cuenta ésto, pregúntale a la persona si desea que tú, u otro familiar o amigo, la acompañe a su cita. Incluso si surgen desafíos en el proceso de la persona para recibir tratamiento debes continuar alentándola a buscar ayuda profesional.

**Si la persona no quiere ayuda profesional (en una situación sin crisis)**

Con cierta frecuencia la persona no desea buscar ayuda profesional. En ocasiones la persona puede no querer ayuda profesional por creer que otros están tratando de lastimarla. Especialmente, si la persona no reconoce que no se encuentra bien, debes ser consciente de que puede resistir activamente los intentos de alentarla a buscar ayuda. Por lo tanto, si la persona no quiere ayuda profesional, en tanto y en cuanto la persona no está en crisis, debes:

- expresar con calma a la persona tu preocupación sobre su elección de no buscar ayuda y las posibles implicancias;
- enfatizar a la persona los beneficios potenciales de obtener ayuda, como el alivio de la ansiedad o de los síntomas que puedan estar perturbándola;
- tratar de mantener una buena relación con la persona, ya que es posible que ella pueda desear la ayuda del asistente en el futuro;
- prepararte para tener varias conversaciones con la persona antes de lograr que ella busque ayuda profesional;
- enfocarte en tratar de encontrar algo con lo cual la persona esté de acuerdo que efectivamente es un problema y, luego, sugerir que la persona busque ayuda para eso. Por ejemplo, si la persona dice que se siente ansiosa en compañía de otras personas, deberías alentarla a buscar ayuda específicamente para ese problema;
- alentarla a hablar con alguien en quien confíe sobre lo que está experimentando;
- tener en cuenta que la persona tiene derecho a rechazar el tratamiento, a menos que cumpla con los criterios para un tratamiento involuntario (es decir, con riesgo cierto e inminente para sí o para terceros);
- conocer cuáles son los indicadores de riesgo para sí mismo o para terceros, para poder actuar en concordancia.

Recuerda que podrías indagar de modo no confrontativo los motivos por los cuales la persona no quiere ayuda profesional y nunca esgrimir el tratamiento u hospitalización involuntarios como una forma de amenaza para que la persona realice o deje de realizar algo.

En el caso que converses tus preocupaciones sobre la persona con un profesional de la salud, debes describir claramente lo que pudiste observar (por ejemplo, exactamente lo que la persona ha estado haciendo y diciendo, dónde y cuándo) para que el profesional tenga toda la información necesaria.

**Alucinaciones y delirios (en una situación sin crisis)**

*Qué son las alucinaciones y los delirios*

Las alucinaciones son percepciones falsas, que nadie más percibe, y que generalmente involucran escuchar voces, o también ver, sentir u oler algo que no es real.

Los delirios son creencias falsas, que pueden ser de persecución, de grandiosidad, de control por parte de otros o de artefactos, etc. Por lo general son particularmente resistentes a las pruebas que demuestran su falsedad y no se suelen resolver por medio de razonamientos o intentos de persuasión.

Que alguien esté con alucinaciones o delirios puede no ser del todo evidente y no necesariamente implica que esté en crisis. En el caso que no esté en crisis, si la persona quiere hablar acerca de sus alucinaciones o delirios, debes escucharla, demostrar empatía y tratar de comprender lo mejor posible lo que está experimentando. Trata de empatizar con cómo se siente la persona con respecto a sus creencias y experiencias, sin establecer ningún juicio sobre el contenido de esas creencias y experiencias.

Pregúntale a la persona si desea hablar o compartir contigo el contenido y contexto de sus delirios o alucinaciones. Pero debes ser consciente de que la experiencia de alucinaciones o delirios de la persona puede hacer que no confíe en otros, incluso en quienes son cercanos. Si la persona muestra desconfianza, no debes potenciar o incrementar esto (por ejemplo, susurrándole algo o hablando con disimulo a otra persona acerca de ella).

Debes saber que si la persona está escuchando voces, puede reaccionar a través de comportamientos, como hablar sola o susurrarse a sí misma, en respuesta a las voces que está escuchando.

Hasta que sepas el contenido y el contexto de los delirios de la persona, es importante tomar recaudos ante reacciones de la persona que pudieran llegar a ser agresivas.

Hay cosas que debes saber que NO tienes que hacer:

- transmitir ningún tipo de acuerdo o desacuerdo con las alucinaciones o los delirios de la persona;
- descartar, minimizar o discutir con la persona sobre sus delirios o alucinaciones;
- actuar alarmado o sorprendido por las alucinaciones o los delirios de la persona;
- reírte o burlarte de los delirios o alucinaciones de la persona;
- alentar a la persona a que intente detener las alucinaciones por medio de su fuerza de voluntad (ya que esto no es posible y, por lo tanto, es inútil decírselo).

*Qué hacer si la persona está experimentando paranoia*

Si la persona está experimentando paranoia, debes:

- en caso de ser necesario darle instrucciones a la persona, asegurarte de que sean simples (por ejemplo, "siéntese y hablemos de ello");
- permanecer con la persona, pero a una distancia que sea cómoda tanto para la persona como para ti;
- decirle a la persona qué vas a hacer antes de hacerlo, por ejemplo, que vas a sacar tu teléfono.

Si la persona desconfía de ti, intenta contar con la ayuda de alguien más. Si observas que la persona se siente en peligro o insegura, deberías tratar de conversar con ella acerca de una forma que a ambos les resulte posible y aceptable para ayudarla a protegerse de la situación.

*En qué consisten los síntomas de paranoia*

La paranoia es un síntoma que consiste en la profunda desconfianza y/o temor hacia otras personas a quienes se les atribuyen otras intenciones que las que pueden ser evidentes para el resto de las personas. Por lo general, el destinatario de esas intenciones es la propia persona (autorreferencia). Pese al convencimiento la persona suele manejarse con particular hermetismo acerca de sus pensamientos. La desconfianza puede estar focalizada con alguna persona, grupo de personas o situación especial y, ocasionalmente, puede ser más generalizada. En ocasiones estos síntomas pueden llegar a configurar un trastorno mental (como el trastorno delirante) o constituir un tipo de trastorno de personalidad (trastorno paranoide de la personalidad), en donde diferentes áreas de la vida de relación de la persona se ven comprometidas.

**Cuando la persona está en crisis**

Se suele considerar que una persona con psicosis está en crisis cuando sus delirios y alucinaciones condicionan significativamente su conducta; el pensamiento puede volverse muy desorganizado y el comportamiento se torna bizarro o disruptivo. Es probable que la persona esté muy inquieta y que sus comportamientos afecten a quienes están cerca. En esos momentos es posible que la persona pueda ponerse en riesgo o poner en riesgo a otras personas. En algunas ocasiones la persona en crisis se comporta de modo agresivo, lo cual puede ir desde la agresión verbal hasta, en casos más severos, la agresión física. En los casos en que una persona con psicosis está en crisis puede requerir medidas de primeros auxilios que son diferentes a cuando la persona con psicosis no está en crisis.

Lo primero que debes tener en cuenta es que debes acercarte con precaución y mantener una distancia segura tanto para no incrementar los temores que pueda estar experimentando la persona como por tu propia seguridad si es que la persona está en un estado psicótico severo o se está comportando agresivamente. Las personas con psicosis no suelen ser agresivas y tienen un riesgo mucho mayor de hacerse daño que otras personas. Sin embargo, ciertos síntomas de psicosis (por ejemplo, alucinaciones visuales o auditivas) pueden hacer que las personas se vuelvan agresivas. En estas circunstancias, debes asegurarte de tener acceso claro y libre de obstáculos a una salida (por ejemplo, quedarte entre la persona y la puerta), tratar de limitar el acceso a los objetos que la persona podría usar para hacerse daño o dañar a otros y, si es seguro hacerlo, intentar eliminar cualquier arma u objeto que pueda usarse como arma en el entorno inmediato de la persona. Hasta que llegue ayuda profesional, si estás solo con la persona, debes llamar a otra persona para que te acompañe. Si hace falta llevar a la persona al hospital, y no sientes que sea seguro hacerlo, debes llamar a los servicios de emergencia.

Es importante que estés atento al modo de comunicarte más allá de la severidad de la crisis de la persona y tener presente que alguien podría actuar en base a sus alucinaciones y delirios aún si no impresiona estar en una crisis tan severa.

Debes capacitarte en evaluar el riesgo de la persona para sí o para terceros. Aun si no te parece que la persona está en riesgo inminente de daño, pero aún estás preocupado por su bienestar, debes preguntarle si hay alguien cercano que pueda acompañarla para estar más segura. Si detectas una amenaza o una advertencia que realiza la persona que está en crisis, y especialmente si se siente perseguida, debes tomarla en serio sin importar si está en estado psicótico severo o agresivo. Si la circunstancia te asusta debes buscar ayuda de inmediato para evitar ponerte en riesgo en cualquier circunstancia que intentes ayudar a una persona con psicosis que está en crisis más allá de la severidad de la misma. Del mismo modo, si la persona tiene un arma o algo que podría usarse como arma no debes acercarte a la persona y debes llamar a los servicios de emergencia inmediatamente. Si la agresión de la persona se sale de control, debes retirarte y llamar a los servicios de emergencia.

Si la persona se vuelve agresiva, debes ser consciente de que la agresión de la persona puede verse exacerbada por cosas que hagas en el intento de ayudarla (por ejemplo, dar participación a la policía).

Si se llama a la policía, debes decirles que la persona puede estar experimentando psicosis y que necesita ayuda de la policía para asegurar el acceso a un tratamiento médico con urgencia y controlar su comportamiento agresivo. Asimismo, debes informar a la policía si la persona tiene o no un arma o algún objeto que podría usar como si fuera un arma.

*Qué deberías evitar cuando la persona está agresiva*

Si la persona se comporta agresivamente, NO debes:

- responder de manera hostil, ni tratar que la persona te haga caso;
- discutir o usar un modo desafiante;
- hacerle demasiadas preguntas, ya que esto puede provocar una actitud defensiva y una mayor ira;
- amenazar a la persona ya que esto puede aumentar el miedo o provocar un comportamiento más agresivo.

Si la persona está en estado psicótico severo debes preguntarle si le gustaría disminuir los estímulos (por ejemplo, apagar la televisión o reducir las luces de la habitación). Si tiene una decisión anticipada / plan de prevención de recaídas, debes seguirlo a menos que la persona se encuentre en riesgo cierto e inminente. Debes tratar de asegurarte que la persona que está en situación de crisis sea evaluada por un profesional de la salud de inmediato. Debes proporcionarle a la persona opciones sobre la búsqueda de ayuda profesional, ya que esto puede darle a la persona un sentido de control (por ejemplo, podrías decirle: "¿quieres ir al hospital conmigo o prefiere que otra persona te acompañe?"). Si llamas a los servicios de emergencia, explícales que la persona necesita ayuda médica de manera urgente.

*Qué son las decisiones anticipadas*

Las decisiones anticipadas, también llamadas “directivas o directrices anticipadas”, son las instrucciones que realiza una persona en pleno uso de sus facultades mentales, con el objeto de que sean cumplidas si, eventualmente en algún momento futuro, no se encuentra en condiciones de expresar su voluntad. Dicho documento, manifestado voluntariamente, tiene carácter legal para entrar en vigor cuando dicha voluntad pueda ser puesta en duda. El fundamento de las decisiones anticipadas es el respeto a la autonomía al amparo de los derechos humanos consagrados en los pactos internacionales y suscriptos por las legislaciones locales, incluidas Argentina y Chile.

Si la persona necesita ir al hospital, debes alentarla a ir voluntariamente. Sin embargo, si la persona va al hospital y es apropiado para la relación que tienen o establecieron, debes tratar de hablar directamente con el médico o el personal de emergencia para proporcionar información relevante acerca de la situación de la persona. Si la persona es trasladada a un centro asistencial u otro (en automóvil, ambulancia o móvil policial), debes acompañarla o procurar que alguien de confianza de la persona lo haga.

*¿A qué se llama “riesgo cierto e inminente”?*

El riesgo cierto e inminente se refiere a la “proximidad de un daño que ya es conocido como verdadero, seguro e indubitable que amenace o cause perjuicio a la vida o integridad física de la persona o de terceros”.

Se supone que tal tipo de riesgo debe ser verificado por medio de una evaluación actual, realizada por el equipo interdisciplinario, que no cabe basar en una etiqueta diagnóstica, en particular si se trata de establecer la necesidad de una internación por motivos de salud mental. Que una persona hubiera estado en riesgo en el pasado, que hubiera sido internada o hubiera recibido un diagnóstico anteriormente no alcanza de ningún modo para presuponer la necesidad de una nueva internación si el riesgo de daño para sí o para otra persona no es lo suficientemente evidente e indubitable, y se puede fundamentar. Su detección por parte de un equipo de salud mental requiere la internación de modo urgente.

Se puede distinguir el “riesgo grave, cierto e inminente” del “riesgo potencial”, aquel que no exige una medida urgente pero que igualmente cabe anticipar y propiciar el accionar profesional para definir una eventual internación que pudiera ser de beneficio para la persona.

**Desescalamiento cuando la persona se encuentra en un estado psicótico grave o se comporta agresivamente**

Se llama “desescalamiento” a la posibilidad de bajar los niveles de crisis de la persona y la severidad de las manifestaciones de los síntomas de psicosis.

*Si la persona se encuentra con síntomas de psicosis y especialmente si se encuentra en estado psicótico severo o se comporta agresivamente*, debes escuchar a la persona (más que hablarle). Igualmente, debes tratar de mantener la calma y no hacer nada que pueda agitar más a la persona. Intenta que la interacción no suba de intensidad (por ejemplo, no hables más alto para intentar asegurarte de ser escuchado por la persona), y hazle saber a la persona que estás allí para ayudarla. Evita el comportamiento nervioso (por ejemplo, hacer movimientos bruscos, hablar rápido, moverte nerviosamente) y trata de no mostrar miedo o ansiedad.

Intenta averiguar con la persona lo que la ayudaría a sentirse segura y en control, y si la persona tiene a alguien en quien confíe (por ejemplo, amigos cercanos o familiares) y, de ser así, procura obtener su ayuda. Debes preguntarle a la persona qué puedes hacer para ayudarla.

*Cuando está en crisis severa o se comporta agresivamente…*

Si hay más de una persona presente, debes alentar a que solo una persona hable a la vez y tratar de crear un espacio alrededor de la persona para que no se sienta abrumada.

Si sospechas que la persona puede estar en una situación de riesgo para sí misma o para otros, debes comunicarte con los servicios de emergencia de inmediato. De la misma manera, si no puedes reducir la situación, debes solicitar asistencia profesional (por ejemplo, un servicio de crisis de salud mental o servicios de emergencia). Y, si los servicios que contactaste desestiman tus preocupaciones acerca de la persona, debes perseverar en intentar buscar apoyo para la persona (por ejemplo, llamando a otro servicio).

Si llamas a los servicios de emergencia debes informarles si la persona está armada o si hay armas accesibles cerca. Y, a su llegada, debes tratar de reunirse con ellos para explicarles la situación antes de acercarse a la persona.

Si llegan otras personas al lugar donde estás conversando con la persona en crisis, debes explicarle a ésta quiénes son, que están allí para ayudar y cómo lo harán.

*En cualquier circunstancia de psicosis…*

Si sospechas que la persona puede estar en una situación de riesgo para sí misma o para otros deberías comunicarte con los servicios de emergencia de inmediato. Si te pones en contacto con un servicio de salud mental, no debes etiquetar a la persona como "psicótica", sino más bien describir los síntomas y problemas actuales en cualquier circunstancia de psicosis, sin importar si está en estado psicótico severo o agresivo. Y si llamas a los servicios de emergencia y la persona ha sido diagnosticada previamente con una enfermedad psicótica, debes explicarlo. Asimismo, debes describirles concisamente tus observaciones específicas sobre el comportamiento y los síntomas de la persona. Deberías informarles si es que crees que la persona corre el riesgo de suicidarse o hacerse daño.

**Cuidados personales para el asistente**

Es común experimentar sentimientos negativos (por ejemplo, conmoción, miedo, tristeza, enojo, frustración) como resultado de apoyar a una persona que tiene síntomas de psicosis. Debes cuidar tu propia salud mental y bienestar. No debes presionarte para encontrar soluciones a todos los problemas de la persona ya que eso te sobrecargará aún más.

Si te estás sintiendo estresado, debes buscar apoyo para ti mismo (por ejemplo, a través de grupos de apoyo y organizaciones, un profesional de salud mental o un amigo que te contenga), mientras mantienes la confidencialidad.

**Propósito de esta guía**

Esta guía de primeros auxilios en salud mental para personas con síntomas de psicosis está destinada a miembros de la comunidad sin necesidad de que tengan experiencia o estudio previo referido a la salud /salud mental.

El rol de la persona capacitada por medio de esta guía es poder ofrecer una ayuda inicial a la persona con síntomas de psicosis hasta cuando ésta reciba la ayuda profesional en el caso de ser necesaria.

**Cómo se desarrolló esta guía**

Esta guía fue desarrollada en base a la opinión de expertas y expertos de la Argentina y de Chile, tanto en cuanto a su formación profesional (médicos generalistas, psiquiatras, psicólogos, psicólogos sociales, etc.) como personas con experiencia vivida en el tema psicosis (propia o como cuidador/a informal).

El equipo de Argentina y de Chile utilizó las guías de primeros auxilios existentes en Australia y realizó la adaptación correspondiente con el asesoramiento de expertos locales. Los detalles de la metodología empleada pueden consultarse en la publicación Agrest M, Encina E, Tapia-Muñoz T, Zamora-Vidal I, Geffner N, Ardila-Gómez S, Alvarado R, Leiderman E, Reavley N. Development of mental health first-aid guidelines for psychosis: a Delphi expert consensus study in Argentina and Chile. (en proceso de publicación).

**Cómo utilizar estas recomendaciones**

Esta guía reúne un conjunto de orientaciones generales. Cada persona es única y es importante adaptar el apoyo que se le entregue a sus necesidades. En este sentido, puede que haya personas que no sean ayudadas aun siguiendo estas recomendaciones o que éstas no sean apropiadas para algunas personas, aun cuando están formuladas en consideración a aspectos culturales de la población chilena y argentina.

Además, debe considerarse muy especialmente que algunas personas presentan junto con sus síntomas de psicosis algún otro tipo de problema de salud o de salud mental que puede requerir abordajes específicos y especializados.

Los recuadros en fondo azul corresponden a las recomendaciones de los expertos locales. En tanto, las secciones de fondo blanco son agregados realizados por los editores de esta guía para complementar o aclarar las sugerencias de los expertos locales.

Estas guías fueron desarrolladas por la Universidad de Palermo, la Universidad de Chile y la Universidad de Melbourne y están protegidas por el derecho de autor. Sin embargo, se pueden usar libremente si no es con fines de lucro.

Se recomienda citar como: Primeros Auxilios en Salud Mental, América Latina. Cómo brindar ayuda a una persona con síntomas de psicosis: Guía de primeros auxilios en salud mental. Universidad de Chile, Universidad de Palermo y Universidad de Melbourne, 2023.

Esta guía se ha elaborado como parte de un conjunto de directrices sobre la mejor manera de asistir a una persona con problemas de salud mental y está disponible en *https://mhfainternational.org/current-mhfa-research/.* El conjunto de recomendaciones de primeros auxilios en salud mental en inglés puede consultarse y descargarse en *https://mhfa.com.au/mental-health-first-aid-guidelines#mhfaesc*

Si tiene dudas respecto de su utilización puede dirigirse a:

Martín Agrest, magrest66@gmail.com

Esteban Encina, esteban2@uchile.cl

Thamara Tapia, thammytapia@gmail.com

Eduardo Leiderman, eduardo.leiderman@gmail.com

Nicola Reavley, nreavley@unimelb.edu.au. +61 3 9035 7628
